# Complex Operator Growth in Dissipative Quantum Systems

**DOI:** 10.3390/e28090953

**Published:** 2026-08-24

**Authors:** Hikaru Wakaura, Taiki Tanimae

**Affiliations:** QIRI (Quantum Integrated Research Institute Inc.), Tokyo 107-0061, Japan; t.tanimae@qiri.co.jp

**Keywords:** operator growth, Krylov complexity, open quantum systems, Lindblad dynamics, quantum chaos, Sachdev–Ye–Kitaev model, non-Hermitian physics

## Abstract

The universal operator-growth hypothesis (OGH) states that, in a closed chaotic system, the Lanczos coefficients grow linearly, $b_{n}≃\alpha n$. We ask how this structure is modified when the system is coupled to a Markovian environment, so that the generator becomes non-Hermitian. Applying the Arnoldi recursion to the vectorized Lindbladian in the infinite-temperature Wightman inner product, we organize the resulting pair of growth rates $\alpha_{C}\equiv \alpha_{R}+i\alpha_{I}$—defined as effective slopes of the sub-diagonal and diagonal Arnoldi coefficients over a pre-registered fit window—around two statements whose logical status we delimit precisely. First, whenever the dissipator acts as $D=-2\gamma \hat{G}$ with $\hat{G}$, a Hermitian grading (all dephasing-type baths), the diagonal obeys the identity Rean=−2γ〈G^〉n: the imaginary rate measures how fast the growing operator accumulates weight in the dissipation channels. Second, we prove a *conditional* parity theorem: if the Hamiltonian, jump operators, and seeds can be made simultaneously real in some basis (an antiunitary condition), then $b_{n}$ is even, and Rean is odd in γ exactly, so $\alpha_{R}$ is renormalized only at $O(\gamma^{2})$, and $\alpha_{I}=2\kappa_{0}\gamma$ follows from closed-system data alone. We exhibit a one-qubit Lindbladian that satisfies the often-assumed generator symmetry $G^{†}\left(\gamma \right)=-G\left(-\gamma \right)$ yet violates parity ($b_{1}=\left|1-\gamma \right|$), showing that the extra condition is essential; all models studied here satisfy it bit-exactly. For large-*q* SYK, these ingredients predict $\alpha_{C}=J-2i\left(q-2\right)\gamma$, whose imaginary part is fixed solely by the interaction range; the first ladder step is exact, and the multi-step increments approach q−2 with system size (1.92±0.04 at N=12, q=4). Under a common fit protocol, the closed-system rates saturate by N=10 ($\alpha_{R}\left(0\right)\to 0.437$, $2\kappa_{0}\to 0.224$). The imaginary rate is not an independent observable at leading order—its content is its *sign*, which resolves how the growing operator meets its environment (opposite for spin chains and SYK).

## 1. Introduction

Operator growth—the progressive delocalization of an initially simple operator under Heisenberg evolution—underlies quantum chaos, thermalization, and the emergence of hydrodynamics [1]. The universal operator-growth hypothesis (OGH) [2] made this quantitative. Expanding the Heisenberg equation of motion in the Krylov basis generated by the Liouvillian $L_{0}=i\left[H,\cdot \right]$, one obtains a semi-infinite hopping chain whose amplitudes, the Lanczos coefficients $b_{n}$, grow linearly, $b_{n}≃\alpha n$ (with a logarithmic correction in one dimension). The single rate α controls the exponential growth of Krylov complexity [3,4], upper-bounds a large class of complexity measures including the out-of-time-order correlator [5,6], and yields $\lambda_{L}\leq 2\alpha$—a bound that is tighter than, and complementary to, the Maldacena–Shenker–Stanford bound $\lambda_{L}\leq 2\pi T$ [7].

Real systems are open. Under Markovian dissipation, the generator of operator dynamics is the (adjoint) Lindbladian, which is *non-Hermitian*, and the symmetric Lanczos algorithm no longer applies: one must use the Arnoldi iteration [8] or a bi-Lanczos scheme [9,10,11], and the OGH has been examined for large-*q* SYK with weak dissipation [12]. These works establish the recursion machinery and compute the coefficients numerically; the one analytic large-*q* SYK rate [12] is extracted from the (real) off-diagonals and stays real, with dissipation entering as a phase. The imaginary part, however, lives in the *diagonal* coefficients $a_{n}$, for which prior open-system studies compute values but, to our knowledge, give no analytic account. What has been missing is a *physical* and *analytic* treatment: what the diagonal measures, how it depends on dissipation, and under what conditions its structure is protected.

Our advance is not that the coefficients are complex—they are, in any non-Hermitian recursion—but that the imaginary rate is pinned down *physically and analytically*: an exact identity identifies it with the operator’s dissipation-grading weight; a *conditional* parity theorem—whose hypothesis we make precise, prove, and delimit with an explicit one-qubit counterexample—fixes its linear-in-γ onset and protects $\alpha_{R}$ to $O(\gamma^{2})$ for the class of models studied here; and large-*q* SYK yields the closed-form prediction $\alpha_{C}=J-2i\left(q-2\right)\gamma$. Two rigorous open-system statements survive en route to a chaos bound (a norm-decay lemma and a locality moment bound), but the chain of inequalities that would deliver $\lambda_{L}\leq 2\alpha_{R}\left(\gamma \right)$ rests on assumptions we do not prove; we state it as an open problem rather than a result. We stress at the outset what the imaginary rate does and does not add: at leading order, it is fixed by closed-system data, $\alpha_{I}=2\kappa_{0}\gamma$, so its content is not an independent number but a *mechanism diagnostic*—its sign resolves how the growing operator meets the environment (accumulating dissipative weight in spin chains, shedding it in SYK)—together with the breakdown of this closed-system predictability once $\alpha_{I}∼\alpha_{R}$.

## 2. Setup: Arnoldi Recursion for the Vectorized Lindbladian

We work at an infinite temperature with the Wightman inner product $\left(A|B\right)=\mathrm{Tr}\left(A^{†}B\right)/\dim$, under which the commutator superoperator [H,·] is Hermitian, and the closed-system generator $L_{0}=i\left[H,\cdot \right]$ is anti-Hermitian. As in all recursion-method studies of open systems, the construction is inner-product dependent; our results are for this standard choice, and their finite-temperature counterparts are examined in Appendix F. The Heisenberg generator of the Lindblad dynamics [13,14] is

(1) $$G\left(O\right)=i\left[H,O\right]+\sum_{\mu }\;\left(L_{\mu }^{†}OL_{\mu }-\frac{1}{2}\left\{L_{\mu }^{†}L_{\mu },O\right\}\right)=L_{0}+D,$$

with $L_{0}=i\left[H,\cdot \right]$ being anti-Hermitian and D being the dissipator. Vectorizing *O* and orthonormalizing the Krylov sequence $\left\{G^{n}O_{0}\right\}$ [15] by the Arnoldi iteration with full reorthogonalization produces an upper-Hessenberg matrix, *h*, whose sub-diagonal $b_{n}=h_{n+1,n}\in R_{\geq 0}$ and diagonal $a_{n}=h_{n,n}\in C$ define

(2) αC≡αR+iαI,bn≃αRn,−Rean≃αIn(n∈[n1,n2]).

Three definitional caveats apply, and we state them up front. (i) At any fixed finite *N*, the grading expectation is bounded, ${\langle \hat{G}\rangle }_{n}\leq N$, so the true n→∞ slopes vanish; a nonzero rate refers to the growth window 1≪n≪N, and the thermodynamic limit must be taken first. The rates are thus *preasymptotic* growth-phase quantities: they characterize the regime before the O(1/γ) crossover at which dissipative oscillations and saturation set in, not a large-*n* asymptote. We, therefore, define $\alpha_{R},\alpha_{I}$ as *effective slopes* over a fit window that is fixed *in advance and common to all system sizes* (n∈[4,18] unless stated), and we report their sensitivity to the window choice in Appendix F. (ii) The two slopes are extracted from two real sequences ($b_{n}$ and −Rean) and bundled into one complex number for compactness; $\alpha_{C}$ is not an eigenvalue, no single observable evolves directly with its magnitude or phase, and it is defined relative to this recursion and inner product (its scheme dependence is quantified in Appendix A); its operational content is the pair $\left(\alpha_{R},\alpha_{I}\right)$ and, on the experimental side, the *t*-odd component of the autocorrelation discussed in Section 5. (iii) For every model studied below the generator and seed can be represented by real matrices; the Krylov vectors then have real *coefficients in that basis* (their components in the computational vectorization are complex), and hence Iman=0 identically—the diagonal *is* its real part (Appendix B).

For γ=0, $G=L_{0}$ is anti-Hermitian, the Hessenberg matrix is tridiagonal, and $b_{n}$ reduce to the OGH Lanczos coefficients with $\alpha_{I}=0$. Numerically, we use a chaotic mixed-field Ising chain, $H=-\sum_{i}Z_{i}Z_{i+1}-g\sum_{i}X_{i}-h\sum_{i}Z_{i}$ with (g,h)=(1.05,0.5), under bulk dephasing $L_{i}=\sqrt{\gamma }\;Z_{i}$; conventions and further models are given in Appendix A and Appendix F. Whenever we report Krylov complexity, we mean

(3) $$K\left(t\right)=\frac{\sum_{n}n\;{\left|\varphi_{n}\left(t\right)\right|}^{2}}{\sum_{n}{\left|\varphi_{n}\left(t\right)\right|}^{2}},\;\varphi_{n}\left(t\right)=(v_{n}|e^{Gt}\left|O_{0}\right),$$

computed from the truncated ($m_{b}=34$) Hessenberg matrix; the explicit normalization is required because the non-unitary evolution does not conserve $\sum_{n}{\left|\varphi_{n}\right|}^{2}$.

## 3. The Complex Growth Rate

### 3.1. An Exact Identity for the Imaginary Rate

For dephasing, the dissipator is diagonal in the Pauli-string basis, $D=-2\gamma \hat{G}$, where $\hat{G}$ is the transverse-weight operator counting the sites on which an operator acts as *X* or *Y*. Decomposing the Arnoldi diagonal as $a_{n}=(v_{n}|L_{0}|v_{n})+(v_{n}\left|D\right|v_{n})$ and using that the first term is purely imaginary ($L_{0}$ anti-Hermitian), while the second is real (D Hermitian) gives the exact identity

**Figure 1:**
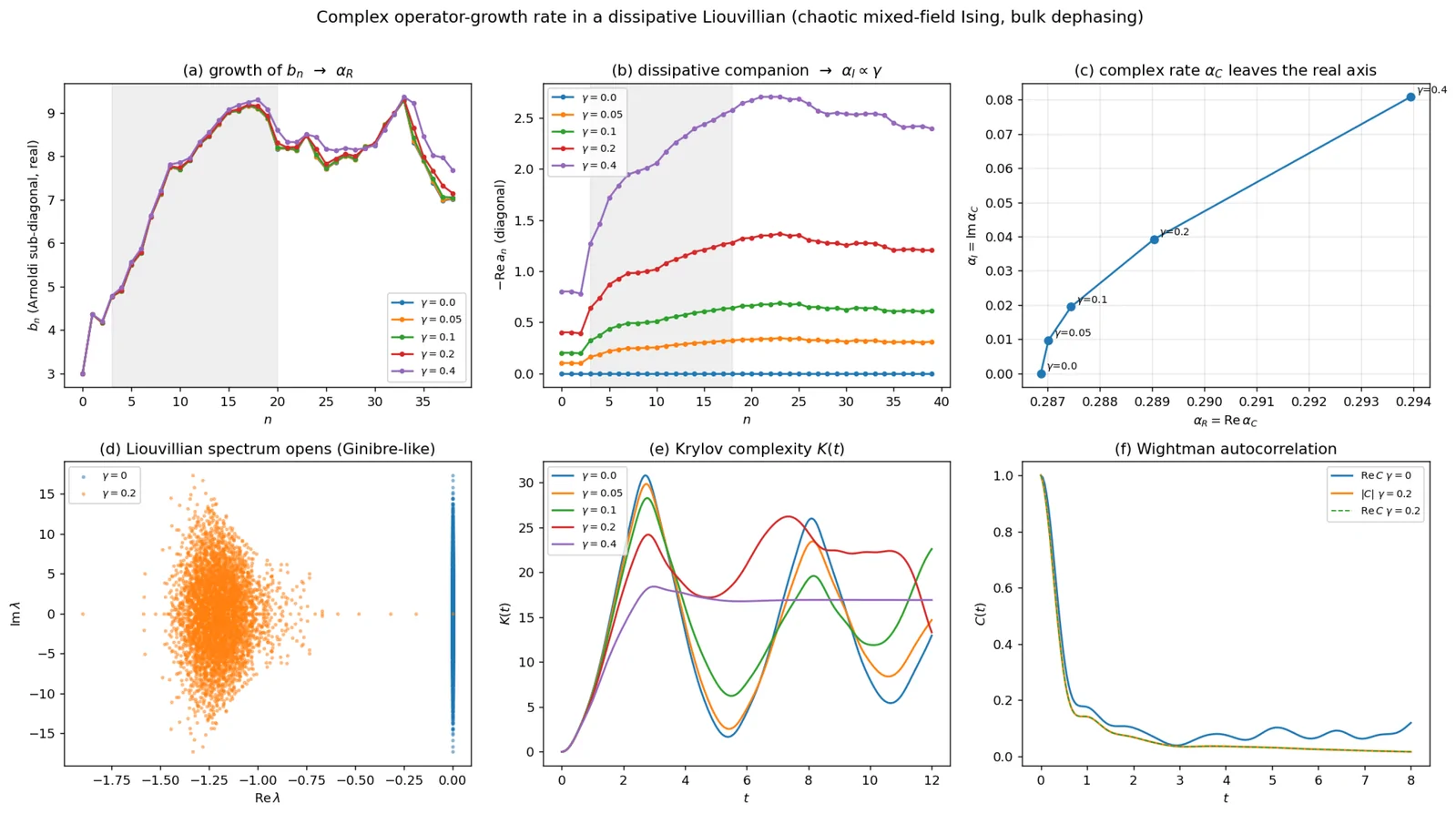
Complex operator-growth rate for a chaotic mixed-field Ising chain (N=6) under bulk dephasing. (**a**) Arnoldi sub-diagonal $b_{n}\to \alpha_{R}n$; (**b**) diagonal companion −Rean→αIn, fanning out ∝γ; (**c**) the complex rate leaves the real axis; (**d**) the Liouvillian spectrum opens from the imaginary axis into the complex plane; (**e**) Krylov complexity, normalized within the truncated Krylov space ($m_{b}=34$); (**f**) Wightman autocorrelation. Grey bands in (**a**,**b**) mark the fit window used for the effective slopes. The uncertainty on all fitted rates is dominated by the window choice; sensitivity bands are given in Appendix F.

(4) Rean=−2γ〈G^〉n,

which is algebraic—it holds for every *H*, γ and *n* (Appendix B); the $10^{-15}$-level numerical check in the companion code validates the implementation, not the physics. We are explicit about its mathematical weight: Equation (4) is the diagonal matrix element of the Hermitian part of G, i.e., a one-line consequence of the decomposition $G=L_{0}+D$—its value lies not in depth but in the *reading* it licenses: the diagonal decay profile is an exact, step-resolved record of how much of the growing operator lies in the channels the environment couples to, $\alpha_{I}=2\gamma \;\sigma_{G}$ with $\sigma_{G}$ the effective slope of ${\langle \hat{G}\rangle }_{n}$ over the fit window (Figure 1a,b).

### 3.2. A Conditional Parity Theorem in the Dissipation Strength

Writing $G\left(\gamma \right)=L_{0}+\gamma \tilde{D}$ with $\tilde{D}$ Hermitian, the generator obeys

(5) $$G^{†}\left(\gamma \right)=-G\left(-\gamma \right)\;\Rightarrow \;\mu_{k}{\left(\gamma \right)}^{*}={\left(-1\right)}^{k}\mu_{k}\left(-\gamma \right),$$

for the moments $\mu_{k}=(O_{0}|G^{k}\left|O_{0}\right)$. It is tempting to conclude that Equation (5) alone forces $b_{n}$ to be even and Rean to be odd in γ—an earlier version of this work did so—but *the inference is false*: for a non-normal generator, the Arnoldi coefficients are functions of the mixed Gram matrix $\Gamma_{jk}=\left(G^{j}O_{0}|G^{k}O_{0}\right)$, which involves ${\left(G^{†}\right)}^{j}G^{k}$ and is *not* determined by the scalar moment sequence. A one-qubit Lindbladian makes this explicit: for H=−Y/2, $L=\sqrt{\gamma }\;Z$ and seed $O_{0}=\left(X+Z\right)/\sqrt{2}$, the generator symmetry in Equation (5) holds exactly, yet the first Arnoldi coefficient is

(6) $$b_{1}\left(\gamma \right)=\left|1-\gamma \right|,$$

manifestly containing a linear term (Appendix C).

Parity is restored by one additional hypothesis. **Theorem (conditional parity).** *Suppose there exists an antiunitary, T, with $T\;G\left(\gamma \right)\;T^{-1}=G{\left(\gamma \right)}^{†}$ and $TO_{0}\propto O_{0}$—equivalently, a basis in which Hamiltonian, jump operators, and seeds are represented by simultaneously real matrices. Then, $v_{n}\left(-\gamma \right)={\left(-1\right)}^{n}Tv_{n}\left(\gamma \right)$, the Gram matrices at ±γ are complex conjugates, and*

(7) $$b_{n}\left(-\gamma \right)=b_{n}\left(\gamma \right),\;a_{n}\left(-\gamma \right)=-a_{n}{\left(\gamma \right)}^{*},$$

*exactly (proof in Appendix C). Consequently, for this class, *$\alpha_{R}\left(\gamma \right)=\alpha_{R}\left(0\right)+c_{2}\gamma^{2}+O\left(\gamma^{4}\right)$, *and *$\alpha_{I}\left(\gamma \right)=2\kappa_{0}\gamma +O\left(\gamma^{3}\right)$. The hypothesis is not exotic: the mixed-field Ising chain with *Z*-dephasing, Majorana-dephased SYK, and the number-dephased Bose–Hubbard trimer all satisfy it, and their parity is verified at machine precision (0.0 for Ising, $4.4\times 10^{-16}$ for SYK; twelve configurations, including deliberately broken ones, in Appendix C). It is, however, a genuine restriction: adding a *Y*-field to the Ising chain with the same seed breaks parity at the $10^{-1}$ level, and the linear-in-γ renormalization of $\alpha_{R}$ returns. The former universality claim is hereby withdrawn.

**Figure 2:**
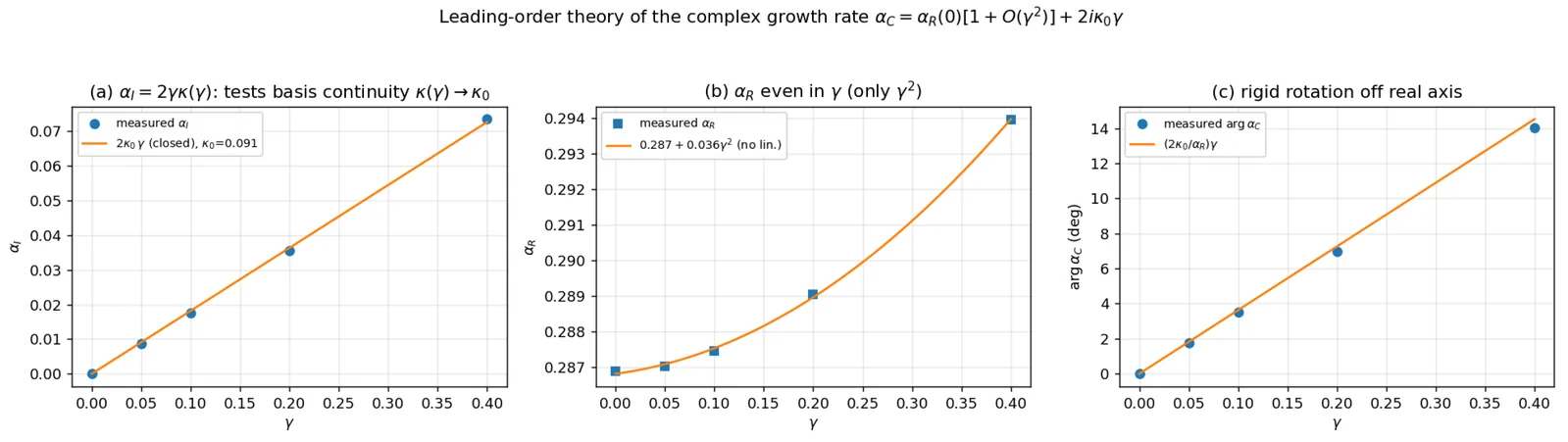
Leading-order structure for the conditional-parity class. (**a**) $\alpha_{I}/\gamma$ versus the closed-system slope $2\kappa_{0}$: within a common window, the identity makes $\alpha_{I}=2\gamma \kappa \left(\gamma \right)$ exact, so this panel tests *basis continuity* $\kappa \left(\gamma \right)=\kappa_{0}+O\left(\gamma \right)$ (5.1% drift across γ≤0.4), not an independent prediction; (**b**) $\alpha_{R}$ even in γ ($c_{2}=0.036$, $c_{1}=0.004$); (**c**) small-angle rotation $\arg\alpha_{C}\approx \left(2\kappa_{0}/\alpha_{R}\right)\gamma$.

There are three quantitative consequences, all under the common fit protocol of Section 2. *(a) Identity versus prediction.* Within any single fit window, the identity in Equation (4) makes $\alpha_{I}\left(\gamma \right)=2\gamma \;\kappa \left(\gamma \right)$ exact by construction (we verify the ratio equals 1 to ten digits); the nontrivial content of Figure 2a is, therefore, *basis continuity*, $\kappa \left(\gamma \right)=\kappa_{0}+O\left(\gamma \right)$—numerically κ(γ)≃0.0869+0.0115γ in the common window, a 5.1% drift across γ≤0.4, with same-window leading-order agreement $\alpha_{I}/\left(2\gamma \kappa_{0}\right)-1=+0.09\%$ at γ=0.05. We no longer describe this as a parameter-free prediction. *(b) Quadratic protection.* In the common window, fitting $\alpha_{R}\left(\gamma \right)=\alpha_{R}\left(0\right)+c_{1}\gamma +c_{2}\gamma^{2}$ over γ≤0.4 gives $c_{1}=0.002$ (consistent with parity) and $c_{2}=0.008$; fixing $c_{1}=0$ as parity dictates gives $c_{2}=0.012$. The value of $c_{2}$ is strongly window- and estimator-dependent (0.01–0.06 across the scan of Appendix F), but its *sign* is stable: $c_{2}>0$, so weak dephasing *increases* the hopping (Figure 2b). *(c) Finite size.* Under the single pre-registered window, for every *N*, the closed-system invariants saturate: $\alpha_{R}\left(0\right)=0.178,0.323,0.402,0.422,0.436,0.437$ and $\kappa_{0}=0.067,0.087,0.105,0.109,0.112,0.112$ for N=5–10; the N=6 used in Figure 1 and Figure 2 sits 26% below the plateau, so those figures illustrate structure, while headline rates should be read from the plateau ($\alpha_{R}\left(0\right)\to 0.437$, $2\kappa_{0}\to 0.224$; Appendix F). In parallel, the Krylov complexity saturates and the Wightman autocorrelation decays with γ (Figure 1e,f), while the Liouvillian spectrum broadens from the imaginary axis into the left half-plane (Figure 1d).

### 3.3. Model Dependence and Large-q SYK

The grading $\hat{G}$—and hence the sign and magnitude of $\alpha_{I}$—is model-dependent, which is the non-universal content of the imaginary rate. For the SYK*_q_* model [16,17] with Majorana dephasing $L_{i}=\sqrt{2\gamma }\;\psi_{i}$, the dissipator reduces, in the size-parity sector of a single-Majorana seed, to the operator co-size: Rean=−2γ(N−〈s〉n), so a growing operator becomes *less* dissipation-sensitive, opposite to the spin chain. The large-*q* size ladder is known—each interaction adds q−2 Majoranas, so ${\langle s\rangle }_{n}=1+\left(q-2\right)n$ [18]—giving $\sigma_{G}=-\left(q-2\right)$. With $\alpha_{R}=J$ [2], we obtain the closed-form *arge-q prediction*

(8) $$\begin{array}{c} \;\alpha_{C}^{\mathrm{SYK}}=J-2i\left(q-2\right)\gamma ,\; \end{array}$$

whose imaginary part is fixed *solely by the interaction range q*, independent of the coupling J. Its logical status is mixed, and we state it plainly: the first ladder step $s_{1}-s_{0}=q-2$ is an operator-algebra identity (exact at any *N*, verified to machine precision), while the identification of subsequent Krylov steps with ladder generations is a large-*q*, large-*N* statement. Disorder-averaged finite-size data support the approach: at *q* = 4 the second increment s_2_ − s_1_ rises from 1.58 ± 0.22 (*N* = 8) through 1.86 ± 0.03 (*N* = 10) to 1.92 ± 0.04 (*N* = 12), approaching *q* − 2 = 2 as finite-size saturation recedes (Appendix D). The exact statements of this paper are Equations (4) and (7) (the latter under its stated hypothesis); Equation (8) is a prediction they jointly motivate.

## 4. Rigorous Partial Results and an Open Problem

Two open-system statements can be made rigorously. First, a norm-decay lemma: $\frac{d}{dt}\left(O|O\right)=2\left(O|D|O\right)=-4\gamma \langle \hat{G}\rangle \left(O|O\right)\leq 0$, so the Wightman norm of any operator is non-increasing under dephasing. Second, a locality moment bound: the generator $G=L_{0}+D$ is a sum of geometrically local, bounded superoperators with per-site strength w≤2J+2γ, and a generalized Lieb–Robinson counting for local Markovian generators [19] gives

(9) $$∥G^{n}{O∥\leq \left(cw\right)}^{n}\;n!\;∥O∥,\;w=2J+2\gamma ,$$

with *c* being a lattice-connectivity constant (Appendix E). Dissipation, therefore, enters the moment growth only through the O(γ) shift of *w*, not through the factorial structure.

In an earlier version, we combined Equation (9) with regularity assumptions to conjecture a dissipative chaos bound $\lambda_{L}^{\mathrm{open}}\leq 2\alpha_{R}\left(\gamma \right)$. We now refrain: (i) the step from factorial moments to linearly growing $b_{n}$ requires a quantitative lower bound on the Krylov Gram determinants that we cannot supply; (ii) the closed-system chain of inequalities relating Krylov growth to the OTOC has no proven non-Hermitian counterpart; and (iii) an operational definition of $\lambda_{L}^{\mathrm{open}}$, together with an actual open-system OTOC computation, would be needed to give the statement empirical content. In addition, since parity gives $c_{2}>0$ for our models, any such bound would be *weaker* than the closed-system one at small γ. Formulating and testing a dissipative chaos bound is, in our assessment, a well-posed open problem, and Equation (9) fixes the one ingredient that dissipation demonstrably does not spoil.

## 5. Discussion

Markovian dissipation splits the operator-growth data into a pair of effective rates, $\alpha_{C}=\alpha_{R}+i\alpha_{I}$, with the imaginary part tied by the exact identity (4) to the operator’s dissipation-grading weight, its γ-dependence fixed—for the antiunitary-symmetric class—by the conditional parity theorem (7), and a closed-form large-*q* SYK prediction (8). The imaginary rate is an environment-resolved diagnostic: $\alpha_{I}$ is the gradient along the Krylov chain of the on-site decay rate −Rean. Its leading order carries no information beyond the closed-system slope $\kappa_{0}$ and the known dissipation strength—that is precisely the content of the parity theorem—so the physics it resolves is its *sign* and the departure from the rigid-rotation regime. For dephasing spins, the decay rate *grows* with depth (transverse weight accumulates, $\alpha_{I}>0$); for Majorana SYK, it *decreases* (a growing operator’s co-size shrinks, $\alpha_{I}<0$)—this is not gain but a negative sensitivity gradient. In neither case does the total norm grow (the norm-decay lemma of Section 4 holds). Two honest limitations frame these results. First, $\alpha_{I}$ is scheme-specific: the corresponding diagonal slope extracted from a bi-Lanczos recursion differs from the Arnoldi value by 50–150% over γ∈[0.05,0.4] (Appendix A), so $\alpha_{C}$ is defined relative to the Arnoldi–Wightman construction, not as a recursion-independent invariant. Second, all rates are window-defined effective slopes (Section 2), with sensitivity quantified in Appendix F.

The construction extends to bosons: in a Bose–Hubbard trimer [20] at fixed total particle number—an energy-bounded, chaotic, *truncation-free* model—the same complex-rate structure appears, with $\alpha_{I}\propto \gamma$ ($\alpha_{I}/\gamma$ drifting by 6% over γ∈[0.05,0.4]), $\alpha_{R}$ even in γ, and the grading identity Reak=−γ2∑i〈(Δni)2〉k holding to $10^{-14}$; both rates converge with $N_{\mathrm{tot}}$ (Appendix F). A single driven Kerr mode, by contrast, is Fock-truncation-limited (Appendix F), so energy boundedness is essential—as it is in finite-energy encodings such as GKP [21]. Experimentally, the coefficients follow from moments of the autocorrelation $C\left(t\right)=(O|e^{Gt}|O)$ via the recursion method; the diagonal $a_{n}$—and hence $\alpha_{I}$—is encoded in the component of C(t) that is *not* even in *t*, absent in the closed system, so the imaginary rate is accessible wherever Lanczos coefficients are already extracted [2]. We caution that this reconstruction is an inference problem in its own right: high-order moments amplify measurement noise rapidly with *n*, so a practical extraction of $a_{n}$ from experimental C(t) will require regularized or otherwise noise-robust estimation; developing such schemes is beyond the scope of this work. Finally, the broadening of the Liouvillian spectrum (Figure 1d) motivates—but we emphasize does not by itself establish—a complex-plane spectral form factor [22] as a dissipative chaos diagnostic.

## Figures and Tables

**Figure A1:**
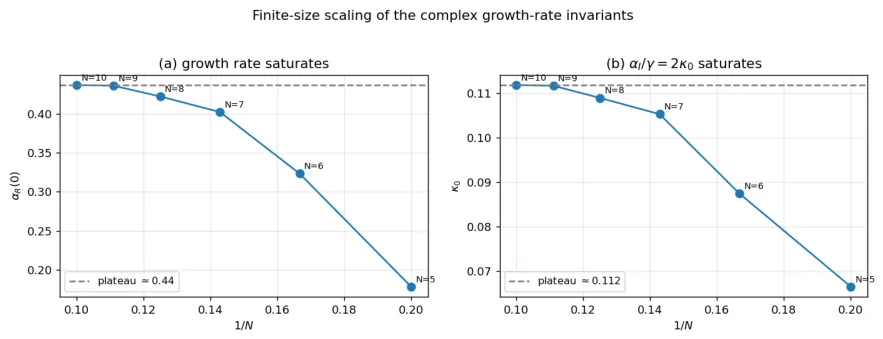
Finite-size scaling of the spin chain (N=5–10, sparse Liouvillian, single pre-registered window (4,18) and m=34 for every *N*): (**a**) $\alpha_{R}\left(0\right)$ and (**b**) $\kappa_{0}$ saturate.

**Figure A2:**
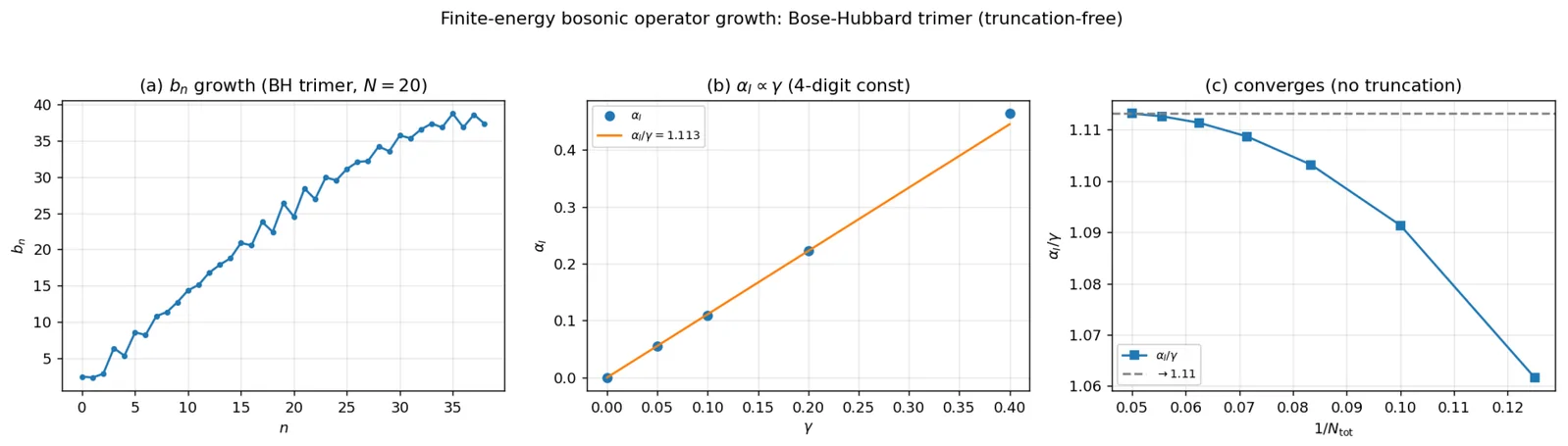
Finite-energy bosonic operator growth (Bose–Hubbard trimer, fixed $N_{\mathrm{tot}}$, no truncation). (**a**) $b_{n}$ grows linearly; (**b**) $\alpha_{I}\propto \gamma$; (**c**) $\alpha_{I}/\gamma$ converges with $N_{\mathrm{tot}}$; the dashed line (arrow in the legend) marks the large-$N_{\mathrm{tot}}$ value 1.11.

**Figure A3:**
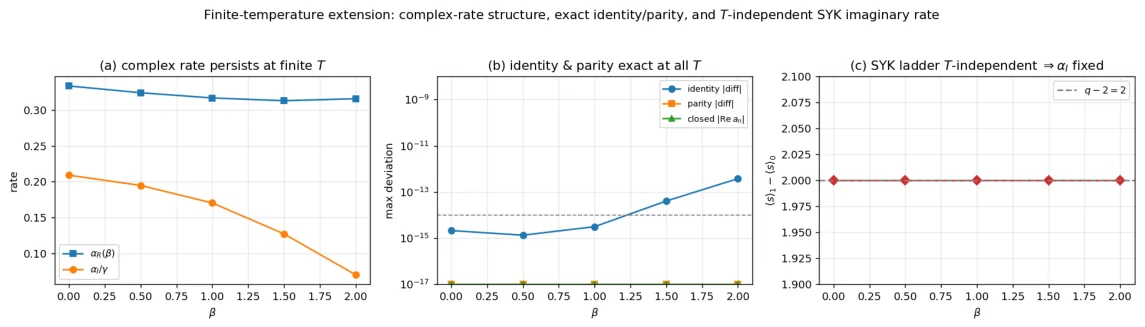
Finite-temperature extension (thermal KMS inner product). (**a**) The complex rate persists: with $\alpha_{R}\left(\beta \right)$ being nearly flat and $\alpha_{I}/\gamma$ decreasing with β. (**b**) The exact identity and the parity theorem ($b_{n}$ even, Rean odd) hold to machine precision at all β (parity and the closed-system Rean sit at the floor). (**c**) The SYK size increment ${\langle s\rangle }_{1}-{\langle s\rangle }_{0}=q-2$ is temperature-independent (red diamonds: measured values at each β; dashed line: q−2=2), so the SYK imaginary rate −2(q−2)γ is fixed by *q* alone.

## Data Availability

The code that reproduces all numerical results and figures reported in this article is openly available under the MIT license at https://github.com/deeptell-inc/complex-operator-growth (accessed on 21 July 2026); a single script (reproduce.sh) regenerates every figure from fixed random seeds. No experimental datasets were generated or analyzed.

## Appendix A. Conventions and the Arnoldi Recursion

We vectorize operators by column stacking, $\mathrm{vec}\left(AXB\right)=\left(B^{T}\;⊗A\right)\;\mathrm{vec}\left(X\right)$, so that the Wightman inner product $\left(A|B\right)=\mathrm{Tr}\left(A^{†}B\right)/\dim$ becomes the Euclidean product on the vectorized space (the 1/dim is an overall scale that cancels in every normalized recursion). The generator is built as $G=i\left(1⊗H-H^{T}\;⊗1\right)+\sum_{\mu }\left[L_{\mu }^{T}\;⊗L_{\mu }^{†}-\frac{1}{2}\left(1⊗L_{\mu }^{†}L_{\mu }+{\left(L_{\mu }^{†}L_{\mu }\right)}^{T}\;⊗1\right)\right]$. The Arnoldi iteration with two passes of modified Gram–Schmidt gives $h_{j,k}=\left(v_{j}|Gv_{k}\right)$, $b_{k}=h_{k+1,k}=∥\;Gv_{k}-\;\sum_{j\leq k}h_{j,k}v_{j}∥$. For γ=0 the anti-Hermiticity of $L_{0}$ makes *h* tridiagonal with real $b_{n}$ and imaginary diagonal, recovering the Lanczos recursion. Although the individual coefficients are basis-dependent, the real rate is comparatively robust across recursion schemes at weak dissipation: $\alpha_{R}$ from the Arnoldi sub-diagonal and from the bi-Lanczos off-diagonals $\sqrt{|b_{n}c_{n}|}$ agree to <0.1% over γ∈[0,0.2] (window (3,20), the original extraction window) (e.g., 0.2869 vs. 0.2869 at γ=0; 0.2890 vs. 0.2892 at γ=0.2), but the agreement degrades to 29% at γ=0.4, so scheme robustness of $\alpha_{R}$ is a weak-dissipation statement. The imaginary rate is *not* scheme-robust: comparing like with like—the slope of −Re of the diagonal coefficients in each scheme—gives (Arnoldi, bi-Lanczos) =(0.0088,0.0133), (0.0176,0.0269), (0.0355,0.0567), (0.0735,0.1820) at γ=0.05,0.1,0.2,0.4: relative differences of 52–147% (panel_supplement.py, part D). $\alpha_{C}$ is therefore reported as an Arnoldi–Wightman-defined quantity throughout.

## Appendix B. Proof of the Exact Identity, Equation (4)

The Arnoldi diagonal is $a_{n}=(v_{n}\left|G\right|v_{n})=(v_{n}|L_{0}|v_{n})+(v_{n}\left|D\right|v_{n})$. Since $L_{0}^{†}=-L_{0}$, the diagonal matrix element $\left(v_{n}|L_{0}|v_{n}\right)$ is purely imaginary; since $D=-2\gamma \hat{G}$ with $\hat{G}={\hat{G}}^{†}⪰0$, the term $(v_{n}\left|D\right|v_{n})=-2\gamma {\langle \hat{G}\rangle }_{n}$ is real. Taking real parts gives Rean=−2γ〈G^〉n for every *n*, γ, and *H*. For dephasing $\hat{G}=\hat{k}$ (transverse weight); for Majorana dephasing in the odd size-parity sector, $D=-2\gamma (N-\hat{s})$ up to the parity factor, giving Rean=−2γ(N−〈s〉n).

For the models studied in this paper, a stronger statement holds: in the Pauli (respectively, Majorana-string, Fock) basis $L_{0}$ is a *real* antisymmetric matrix, and the seed is real, so every Krylov vector has real coefficients in that basis (verified: maximum imaginary Pauli-basis coefficient $1.6\times 10^{-16}$, while computational-vectorization components are complex), $(v_{n}|L_{0}|v_{n})=0$ identically, and Iman=0 at machine precision (at the $10^{-13}$ level; max $1.2\times 10^{-13}$ across γ). The diagonal is then not merely constrained by Equation (4)—it *equals* $-2\gamma {\langle \hat{G}\rangle }_{n}$. The “complex” rate $\alpha_{C}$ is, in this sense, a bundling of two real slopes, as stated in Section 2.

## Appendix C. The Conditional Parity Theorem: Counterexample, Proof, and Verification

**Moment symmetry.** With $G\left(\gamma \right)=L_{0}+\gamma \tilde{D}$, $L_{0}^{†}=-L_{0}$ and ${\tilde{D}}^{†}=\tilde{D}$, one has $G^{†}\left(\gamma \right)=-L_{0}+\gamma \tilde{D}=-G\left(-\gamma \right)$, hence $\mu_{k}{\left(\gamma \right)}^{*}={\left(-1\right)}^{k}\mu_{k}\left(-\gamma \right)$ for the moments $\mu_{k}=(O_{0}|G^{k}\left|O_{0}\right)$—Equation (5).

**Why moments do not suffice.** For γ=0, the generator is anti-Hermitian, and the Krylov Gram matrix is a Hankel form in the moments, $\Gamma_{jk}=\left(L_{0}^{j}O_{0}|L_{0}^{k}O_{0}\right)={\left(-1\right)}^{j}\mu_{j+k}$; Lanczos coefficients are then functions of $\left\{\mu_{k}\right\}$, which is the textbook closed-system statement. For γ≠0, the generator is non-normal, $\Gamma_{jk}=(O_{0}|{\left(G^{†}\right)}^{j}G^{k}\left|O_{0}\right)$ cannot be reduced to powers of G alone, and the moment symmetry constrains the diagonal of Γ but not the recursion. **Counterexample.** Take the one-qubit Lindbladian H=−Y/2, $L=\sqrt{\gamma }\;Z$, seed $O_{0}=\left(X+Z\right)/\sqrt{2}$. In the (X,Z) Pauli sector, $L_{0}=\left(\begin{array}{cc} 0 & 1\\ -1 & 0 \end{array}\right)$, $\tilde{D}=\mathrm{diag}\left(-2,0\right)$, a two-line computation gives $a_{0}=-\gamma$, and $b_{1}=\left|1-\gamma \right|$: Rea0 is odd, but $b_{1}$ has an exact linear term—Equation (6). The generator symmetry and all hypotheses of the naive argument hold ($∥G^{†}\left(\gamma \right)+G\left(-\gamma \right)∥=0$ exactly).

**Proof of the conditional theorem.** Assume an antiunitary *T* with $TG\left(\gamma \right)T^{-1}=G{\left(\gamma \right)}^{†}$ and $TO_{0}=e^{i\theta }O_{0}$. Combine with the generator symmetry, $TG\left(\gamma \right)T^{-1}=-G\left(-\gamma \right)$, so $G{\left(-\gamma \right)}^{k}O_{0}={\left(-1\right)}^{k}T\;G{\left(\gamma \right)}^{k}O_{0}$ (up to the seed phase). Since *T* is antiunitary, inner products conjugate: $\Gamma_{jk}\left(-\gamma \right)={\left(-1\right)}^{j+k}\bar{\Gamma_{jk}\left(\gamma \right)}$. The Arnoldi recursion is applied to a Gram matrix, and its (sign-conjugated) complex conjugate produces $v_{n}\left(-\gamma \right)={\left(-1\right)}^{n}e^{i\theta }Tv_{n}\left(\gamma \right)$, sub-diagonal norms $b_{n}\left(-\gamma \right)=b_{n}\left(\gamma \right)$ (norms are conjugation-invariant), and diagonals $a_{n}\left(-\gamma \right)=(v_{n}\left|G\left(-\gamma \right)\right|v_{n}{)}_{-\gamma }=-\bar{a_{n}\left(\gamma \right)}$: with Rean being odd and Iman being even. The hypothesis is equivalent to the existence of a basis in which *H*, all jump operators, and $O_{0}$ are simultaneously real matrices (*T* = complex conjugation in that basis).

**Verification across twelve configurations** (parity_conditions.py; all γ=0.2, N=4 Ising unless noted; entries are $\max_{n}\left|b_{n}\left(+\gamma \right)-b_{n}\left(-\gamma \right)\right|$). Parity holds bit-exactly whenever the hypothesis is satisfiable and breaks at $O(10^{-1})$ whenever it is not: standard *H* with seeds $X_{c}$, $Y_{c}$, $\left(X_{c}+Z_{c}\right)/\sqrt{2}$, $\left(X_{c}+iY_{c}\right)/\sqrt{2}$ all give 0.0; the mixed seed $\left(X_{c}+Y_{c}\right)/\sqrt{2}$ (not simultaneously realizable) gives $1.7\times 10^{-1}$; adding a *Y*-field (f=0.7) with seed $X_{c}$ gives $2.3\times 10^{-1}$, while the same *H* with seed $Z_{c}$ or with the rotated seed $\cos\varphi X_{c}+\sin\varphi Y_{c}$, φ=arctan(f/g) (both real in a rotated frame) restores $1.8\times 10^{-15}$, and the sign-flipped rotated seed $\cos\varphi X_{c}-\sin\varphi Y_{c}$ (not co-realizable) breaks again at $1.9\times 10^{-1}$; SYK (N=10, Majorana dephasing) gives $4.4\times 10^{-16}$; the one-qubit counterexample gives 0.4, and rotating its seed to $\left(Y+Z\right)/\sqrt{2}$ (real after a frame change) restores 0.0. The necessity of the hypothesis is not proven, but in all four tested configurations where it fails, parity fails.

**Quadratic coefficient.** In the common window (4,18), the two-parameter fit gives $\left(c_{1},c_{2}\right)=\left(0.002,0.008\right)$, and the parity-constrained fit ($c_{1}\equiv 0$) gives $c_{2}=0.012$; in the window (3,20), the same two estimators give (0.004,0.036) and 0.045, and the pointwise estimator $\left[\alpha_{R}\left(\gamma \right)-\alpha_{R}\left(0\right)\right]/\gamma^{2}$ ranges over 0.044–0.059. The spread is thus dominated by the choice of window *and* estimator (a floated $c_{1}$ absorbs curvature and lowers $c_{2}$ by tens of percent); values quoted in earlier drafts (0.053–0.059) are pointwise estimates in the (3,20) window. Only the sign and order of magnitude of $c_{2}$ are protocol-robust.

## Appendix D. SYK Size Ladder and the Large-q Closed Form, Equation (8)

**Imaginary part.** We use the established large-*q* SYK size ladder [18]: each *q*-body interaction vertex adds exactly q−2 Majoranas, so the generation-*k* operator has size s=1+(q−2)k [RSS Equation (3.4)], and the mean size grows as $\langle s\left(t\right)\rangle =\cosh\left(2J^{\left(t\right)}\right)$. Identifying the generation with the Krylov index (each Liouvillian step applies one vertex), ${\langle s\rangle }_{n}=1+\left(q-2\right)n$, so 〈s〉=1+(q−2)K(t) and $\sigma_{G}=-d\langle s\rangle /d\langle n\rangle =-\left(q-2\right)$ exactly. Combined with our identity (4) (co-size sector, $\hat{G}=N-\hat{s}$), this gives $\alpha_{I}=-2\left(q-2\right)\gamma$. We independently confirm that the ladder at finite *q*: $O_{1}\propto \left[H_{q},\psi_{0}\right]$ has size exactly q−1 (writing a *q*-body term as $\psi_{0}M_{q-1}$, $\left[\psi_{0}M,\psi_{0}\right]=\frac{1}{2}M\left(1-{\left(-1\right)}^{q-1}\right)=M$ for even *q*), so $s_{1}-s_{0}=q-2$ exactly—verified at machine precision, 2.0000±0.0000 over five disorder realizations at every *N*. The second increment is finite-size-limited but approaches q−2 as *N* grows. All size expectations are computed directly on the *orthogonalized Arnoldi vectors* (${\langle s\rangle }_{n}=(v_{n}|\hat{s}\left|v_{n}\right)$), not on raw powers $G^{n}O_{0}$, so any Krylov-index/generation mixing is included in the data, and the limits are taken in the order N→∞ first and then with *n*, at a fixed *q* (the ladder is a closed-system operator-algebra statement, so γ enters only through the identity): at q=4, $s_{2}-s_{1}=1.58\pm 0.22$, 1.86±0.03, 1.92±0.04 for N=8,10,12 (mean ± std over five disorder realizations; panel_supplement.py, part C). At a fixed *N*, the early-window slope *decreases* with *q* (the s≤N ceiling is reached in fewer steps), so the approach to the ladder must be read in *N* at fixed a *q*, not in *q* at a fixed *N*.

**Real part.** $\alpha_{R}=J$ is confirmed by applying the recursion method in high precision (60 digits) to the exact large-*q* autocorrelation $C(t)=1+\frac{2}{q}$ ln sech (Jt)—whose singularity at Jt=iπ/2 sets an analyticity strip of width π/2J— giving $b_{n}≃J(n-\frac{1}{2})$, so $b_{n}/n\to J$.

## Appendix E. Locality Moment Bound and Norm Decay, Equation (9)

**Locality.**  $G=\sum_{a}g_{a}$ with each $g_{a}$ geometrically local and bounded; the per-site strength is w≤2J+2γ, the dissipator adding on-site terms of strength ≤2γ. **Moment bound.** Expanding $G^{n}O=\sum g_{a_{1}}\cdots g_{a_{n}}O$, a term is nonzero only if each factor overlaps the support built by the previous ones; in *D* spatial dimensions, the number of such ordered sequences is bounded by n! times a geometric (connectivity) factor $c^{n}$, and each factor contributes ≤w, giving $∥G^{n}{O∥\leq \left(cw\right)}^{n}n!∥O∥$—the superoperator form of a generalized Lieb–Robinson bound for local Markovian generators [19]. Hence, $|\mu_{n}{|\leq ∥O∥}^{2}{\left(cw\right)}^{n}n!$; D enters only by shifting w→w+2γ, not the factorial structure, so the Taylor series of $C\left(t\right)=\sum_{n}\mu_{n}t^{n}/n!$ converges in a disk of radius $1/\left(cw\right)\geq \tau_{0}-O\left(\gamma \right)$ around the origin. **Norm decay.** $\frac{d}{dt}\left(O|O\right)=(O|G+G^{†}\left|O\right)=2\left(O|D|O\right)=-4\gamma \langle \hat{G}\rangle \left(O|O\right)\leq 0$.

We deliberately stop here. Earlier drafts continued with “factorial moments + Gram non-degeneracy ⇒$b_{n}\leq Cn$⇒ $\lambda_{L}\leq 2\alpha_{R}\left(\gamma \right)$”; that chain requires a quantitative lower bound on $\det\Gamma_{n}$, an extension of disk convergence to a strip along the real axis, and a non-Hermitian counterpart of the closed-system Krylov–OTOC inequalities—none of which we can currently supply (Section 4).

## Appendix F. Robustness, Finite-Size Scaling, and the Continuous- Variable Caveat

**Fit-window sensitivity (pre-registered protocol).** Because the N=6 sequences saturate near n∼17 and oscillate beyond, every quoted rate is a window-defined effective slope (Section 2). Scanning 33 windows $\left(n_{1},n_{2}\right)$ with $n_{1}\in \left[3,10\right]$, $n_{2}\in \left[14,28\right]$ at N=6 yields $\alpha_{R}\left(0\right)\in \left[-0.02,0.40\right]$ and $\alpha_{I}/\gamma \in \left[0.09,0.22\right]$ at γ=0.2 (panel_supplement.py, part A): the window choice, not statistical noise, dominates the uncertainty of any single quoted number. All headline values in this paper, therefore, use the single pre-registered window (4,18) common to every *N* and every model, and finite-size convergence below is the evidence that the protocol measures a stabilizing quantity.

**Robustness and scope.** All quantitative results are at an infinite temperature for the mixed-field Ising chain with bulk dephasing. Across chaotic parameters and seeds, the rates vary—at γ=0.2, window (4,18): $\left(\alpha_{R},\alpha_{I}/\gamma \right)=\left(0.324,0.177\right)$ [seed *X*], (0.325,0.256) [seed *Z*], (0.329,0.204) [g=0.9,h=0.8]—confirming that the *magnitude* $\sigma_{G}$ is non-universal (operator- and model-dependent, exactly as α is in the closed OGH), while the *structure* ($\alpha_{I}\propto \gamma$, the conditional parity, the identity) is common to the class. At the near-integrable point g=1,h=0 the linear growth and, hence, both rates collapse ($\alpha_{R}\approx 0$), as expected.

**Finite temperature.** Using the thermal (KMS) inner product ${\left(A|B\right)}_{\beta }=\mathrm{Tr}(\rho^{1/2}A^{†}\rho^{1/2}$B)/Z, under which $L_{0}$ is anti-Hermitian (so the closed diagonal stays imaginary, Rean=0 to machine precision), the complex-rate structure persists (Figure A3): $\alpha_{I}/\gamma =0.209,0.195,0.170$ for β=0,0.5,1.0, and the identity Rean=−2γ〈k^〉n holds to $10^{-15}$ with the *thermal* transverse-weight expectation—so it is not an infinite-temperature artifact. We are explicit about what does *not* extend: with $\rho =e^{-\beta H}/Z$ and $[L_{i},H]\neq 0$, the dephasing dissipator is *not* self-adjoint in the KMS product ($∥{\tilde{D}}^{{*}_{\beta }}-\tilde{D}∥\approx 2.5\times 10^{2}$ at N=4, β=0.5), and consequently, the naive generator symmetry $G^{{*}_{\beta }}\left(\gamma \right)=-G\left(-\gamma \right)$ fails as well ($∥G^{{*}_{\beta }}\left(\gamma \right)+G\left(-\gamma \right)∥\approx 49$). Parity nevertheless holds, and provably so: the conditional theorem of Appendix C extends verbatim to any inner product whose metric is a *real* matrix in a basis where $L_{0}$ is purely imaginary, $\tilde{D}$ is real, and the seed is real—complex conjugation *T* then satisfies $TG\left(\gamma \right)T^{-1}=-G\left(-\gamma \right)$ and leaves the metric invariant, so the weighted Gram matrices at ±γ are complex conjugates. The KMS metric is real for a real *H*, hence $b_{n}\left(-\gamma \right)=b_{n}\left(\gamma \right)$ and Rean(−γ)=−Rean(γ)*exactly*—observed bitwise (0.0) at every β: the entire leading-order structure ($\alpha_{R}$ even, $\alpha_{I}\propto \gamma$) extends to finite temperature. Likewise, [ρ,H]=0 makes $L_{0}$ KMS-anti-self-adjoint (verified: Rean=0 at γ=0 for every β), which is all the identity argument needs. The locality moment bound, Equation (9), is temperature-independent. For SYK, the imaginary rate is itself *T*-independent: the size ladder s=1+(q−2)k is an operator-algebra fact (numerically, $s_{1}-s_{0}=q-2$ to machine precision at β=0,0.5,1.0), so $\alpha_{C}^{\mathrm{SYK}}\left(\beta \right)=\alpha_{R}\left(\beta \right)-2i\left(q-2\right)\gamma$ with *all* temperature dependence in $\alpha_{R}\left(\beta \right)$, which interpolates from J(β→0) to π/β(β→∞, maximal chaos).

**Non-Hermitian jumps.** For amplitude damping, the dissipator is not self-adjoint in the Wightman product, so neither the grading identity nor the conditional parity theorem applies; what follows is a purely *numerical* observation. For damping on every site, $L_{i}=\sqrt{\gamma }\;\sigma_{i}^{-}$: $\alpha_{I}/\gamma =0.095,0.095,0.094,0.091$ for γ=0.05,0.1,0.2,0.4 (nearly constant), with $\alpha_{R}$ rising only quadratically from 0.287 to 0.293 (panel_supplement.py, part B). The clean grading identity is specific to dephasing (where D is diagonal in the Pauli basis); for general jumps, Rean remains the self-adjoint part of the diagonal but is not a single grading expectation.

**Finite size.** Using a sparse Liouvillian, we reach N=5–10 (operator space up to $4^{10}\approx 10^{6}$; Figure A1). Under the single pre-registered window (4,18) applied identically at every *N*—no per-*N* tuning—both invariants saturate: $\alpha_{R}\left(0\right)=0.178,0.323,0.402,0.422,0.436,0.437$ and $\kappa_{0}=0.067,0.087,0.105,0.109,0.112,0.112$ for N=5,…,10 (panel_supplement.py, part E), hence $\alpha_{I}/\gamma \to 2\kappa_{0}\approx 0.224$; the change from N=9 to N=10 is 0.2%. The N=6 used in the main-text figures sits 26% below the plateau, so plateau values are the headline rates. The finite-size correction is not a clean 1/N, so we quote the plateau rather than a linear extrapolation.

**Bosonic (continuous-variable) realization.** We use a Bose–Hubbard trimer, $H=-J\sum_{\langle ij\rangle }\left(a_{i}^{†}a_{j}+h.c.\right)+\frac{U}{2}\sum_{i}n_{i}\left(n_{i}-1\right)$ on a triangle, at fixed total particle number $N_{\mathrm{tot}}$ (Hilbert dimension $\left(N_{\mathrm{tot}}+1\right)\left(N_{\mathrm{tot}}+2\right)/2$, a *complete* sector with no truncation), number-dephasing jumps $L_{i}=\sqrt{\gamma }\;n_{i}$, mean-field scaling $U=u/N_{\mathrm{tot}}$, and seed $n_{0}-n_{1}$. Number dephasing is diagonal in the Fock basis with grading $\hat{G}=\frac{1}{4}\sum_{i}{\left(\Delta n_{i}\right)}^{2}$ (coherence order), so Reak=−γ2∑i〈(Δni)2〉k, verified to $10^{-14}$. The complex-rate structure is clean (Figure A2): $\alpha_{I}=0$ at γ=0, $\alpha_{I}/\gamma$ is constant to four digits, $\alpha_{R}$ is even, and $\alpha_{I}/\gamma$ converges to ≈1.11 as $N_{\mathrm{tot}}\to 20$. For contrast, a single driven-dissipative Kerr mode with photon loss has no bounded sector, and we could not obtain truncation-converged rates there, which is why energy boundedness is essential.
